# Predictive action tracking without motor experience in 8-month-old infants

**DOI:** 10.1016/j.bandc.2016.09.010

**Published:** 2016-11

**Authors:** C.C.J.M. de Klerk, V. Southgate, G. Csibra

**Affiliations:** a Centre for Brain and Cognitive Development, Birkbeck College, University of London, United Kingdom; b Institute of Psychology, University of Copenhagen, Denmark; c Cognitive Development Center, Central European University, Budapest, Hungary

**Keywords:** Action prediction, Predictive action tracking, Infant development, Motor experience, Sensorimotor alpha, EEG

## Abstract

A popular idea in cognitive neuroscience is that to predict others’ actions, observers need to map those actions onto their own motor repertoire. If this is true, infants with a relatively limited motor repertoire should be unable to predict actions with which they have no previous motor experience. We investigated this idea by presenting pre-walking infants with videos of upright and inverted stepping actions that were briefly occluded from view, followed by either a correct (time-coherent) or an incorrect (time-incoherent) continuation of the action (Experiment 1). Pre-walking infants looked significantly longer to the still frame after the incorrect compared to the correct continuations of the upright, but not the inverted stepping actions. This demonstrates that motor experience is not necessary for predictive tracking of action kinematics. In a follow-up study (Experiment 2), we investigated sensorimotor cortex activation as a neural indication of predictive action tracking in another group of pre-walking infants. Infants showed significantly more sensorimotor cortex activation during the occlusion of the upright stepping actions that the infants in Experiment 1 could predictively track, than during the occlusion of the inverted stepping actions that the infants in Experiment 1 could not predictively track. Taken together, these findings are inconsistent with the idea that motor experience is necessary for the predictive tracking of action kinematics, and suggest that infants may be able to use their extensive experience with observing others’ actions to generate real-time action predictions.

## Highlights

• Can infants predictively track the kinematics of actions outside their motor repertoire?
• Pre-walking infants predictively tracked upright, but not inverted stepping actions.
• Sensorimotor cortex was activated more when infants observed upright stepping actions.
• Motor experience is not necessary for predictive tracking of action kinematics.

## 1 Introduction

From the moment they come into the world, infants are surrounded by people who are performing actions that they are unable to perform themselves. How infants form real-time predictions about these actions is an important question considering the crucial role action prediction plays in joint action, cooperation, and collaboration (Sebanz & Knoblich, 2009). It has often been suggested that to predict others’ actions, observers need to map the actions onto their own motor repertoire (e.g. Knoblich and Flach, 2001, Neal and Kilner, 2010, Springer et al., 2011, Wilson and Knoblich, 2005). The results of several behavioural and neurophysiological studies indeed suggest that the motor system plays a functional role in action prediction in both infants and adults. For example, performing incongruent actions (Springer et al., 2011) or being restricted to act (Ambrosini, Sinigaglia, & Costantini, 2012) during action observation has been shown to interfere with participants’ prediction abilities, and eye-tracking studies have demonstrated a relationship between infants’ developing motor repertoire and their ability to predict other people’s actions (Cannon and Woodward, 2012, Cannon et al., 2012, Falck-Ytter et al., 2006, Gredebäck and Kochukhova, 2010, Gredebäck et al., 2009, Kanakogi and Itakura, 2011, Stapel et al., 2016). Furthermore, neurophysiological studies suggest that the motor system is recruited whenever observers are generating action predictions (Kilner et al., 2004, Ramnani and Miall, 2004, Southgate et al., 2010, Southgate et al., 2009, Southgate and Vernetti, 2014). For example, Southgate et al. (2009) found that after observing a few repetitions of goal-directed reaching actions, 9-month-old infants began to show sensorimotor cortex activation prior to the onset of the action, suggesting that they were anticipating the impending action.

Although these studies demonstrate that the motor system plays a role in action prediction, there is still considerable debate concerning the precise nature of this role. The link between motor system activation and action prediction has led some to suggest that motor experience is crucial for action prediction. This account proposes that observers automatically activate the motor representations of the actions they observe, which in turn allows them to understand and predict the goal of those actions (Flanagan and Johansson, 2003, Green et al., 2014, Möller et al., 2015, Rat-Fischer et al., 2014, Rizzolatti et al., 2001). Thus, it follows from this account that infants should be unable to predict actions that are outside their motor repertoire because they lack access to a corresponding functional motor representation.

Alternative accounts propose that actions are first interpreted at some level of visual and/or conceptual analysis before they are transformed into motor code (e.g., Csibra, 2007, Jacob, 2008, Kilner et al., 2007). The reconstruction of the motor commands needed to perform the action allows the observer to predict the visual consequences of the action by invoking the forward model (Wolpert and Flanagan, 2001, Wolpert and Ghahramani, 2000), which is normally used to predict the sensory effects of the observer’s own actions (Csibra, 2007). Furthermore, this account suggests that when the observer is unable to perform the observed action, he or she may activate the motor program for an action that could bring about similar effects (Csibra, 2007, Schubotz, 2007). Thus, while the first account claims that activation of a corresponding motor representation is a prerequisite for recognising the action and predict its further course (Flanagan and Johansson, 2003, Green et al., 2014, Rizzolatti et al., 2001), the second account advocates that visual information alone is sufficient to support action understanding, which in turn allows the observer to use the motor system to predict how the action will unfold.

In support of this latter account, recent studies have demonstrated that infants *can* predict actions irrespective of whether these actions can be mapped onto a corresponding functional motor representation (Biro, 2013). Furthermore, it has been shown that the motor system plays a role in the prediction of such non-executable actions (Southgate & Begus, 2013). However, as these studies used self-propelled objects and claws, it is currently unclear whether infants are also able to predict *human* actions that are outside their motor repertoire. Another limitation of previous work on action prediction in infancy is that the goal and the path of the observed actions are often conflated, making it unclear whether prediction of the goal state or movement path was measured (e.g., Cannon et al., 2012, Falck-Ytter et al., 2006, Southgate and Begus, 2013).

In the current study we aimed to address these issues, and advance the debate by focusing specifically on infants’ ability to predict the *kinematics* of human actions and by asking whether there is a need for motor competence. Based on previous work with adult participants, we hypothesised that infants may be able to use their previous visual experience to support the real-time prediction^1^ of actions that are outside their motor repertoire, and that they might activate motor programs for actions that can bring about similar effects when doing so. For example, Cross, Stadler, Parkinson, Schütz-Bosbach, and Prinz (2013) demonstrated that visual training improved adult participants’ ability to predict intransitive actions they had never performed before (such as the movements of a gymnast or wind-up toy) and that this prediction process was associated with activation of the motor system. In this study, there was no goal object or location to guide participants’ predictions. Nevertheless, visual experience with the actions may have allowed participants to extract information about the temporal dynamics of the observed actions, enabling them to activate motor programs for alternative actions with similar dynamics to generate predictions about how the actions would unfold (Schubotz, 2007). We hypothesised that infants, who spend a considerable amount of time simply watching the actions of people around them, may also be able to use visual experience to support the real-time prediction of actions that they have no motor experience with (Hunnius and Bekkering, 2010, Hunnius and Bekkering, 2014), possibly by activating motor programs for actions with similar temporal dynamics. The present study aimed to investigate this idea by testing pre-walking infants’ ability to differentiate between walking actions that continued either correctly or incorrectly after a brief occlusion period, and the neural mechanisms supporting this ability.

## 2 Experiment 1: Looking time study

To measure predictive action tracking in infants, we adopted a paradigm that has previously been used to investigate real-time action prediction of point-light stimuli in adult participants (Graf et al., 2007, Parkinson et al., 2012, Sparenberg et al., 2012). Infants were presented with videos of upright and inverted (upside-down) infant stepping actions that were briefly occluded from view, followed by either a correct (time-coherent) or an incorrect (time-incoherent) continuation of the action. We then measured infants’ looking times to static test postures after correctly versus incorrectly continued actions. We used infant stepping stimuli because a previous study suggested that visual experience with this type of action might trigger predictive processes (de Klerk, Johnson, Heyes, & Southgate, 2015). We used inverted stepping actions as control stimuli to check whether extrapolation from the movement prime would be sufficient to elicit predictive responses. We hypothesised that infants do not necessarily need active motor experience, but can rely on their previous observational experience with human actions to generate real-time action predictions. Therefore, we predicted that pre-walking infants would be able to distinguish between the correct and incorrect continuations of visually familiar upright stepping actions (as indicated by longer looking times to incorrectly continued actions compared to correctly continued actions), but not of unfamiliar inverted stepping actions.

### 2.1 Method

#### 2.1.1 Participants

The final sample consisted of 24 pre-walking 8-month-old infants (M = 245 days; range 228–268 days). An additional nine infants were tested but excluded because they did not provide enough trials for analyses due to fussiness (N = 5), experimental error (N = 1) or failure to engage with the stimuli (N = 3). Two more infants were excluded because they did not fulfil the inclusion criteria: one infant was born 5 weeks pre-term and another was already cruising (walking while holding on to furniture). All included infants were born full-term, healthy and with normal birth weight. Written informed consent was obtained from the infant’s caregiver prior to the start of the experiment.

#### 2.1.2 Stimuli

**Fig. 1:**
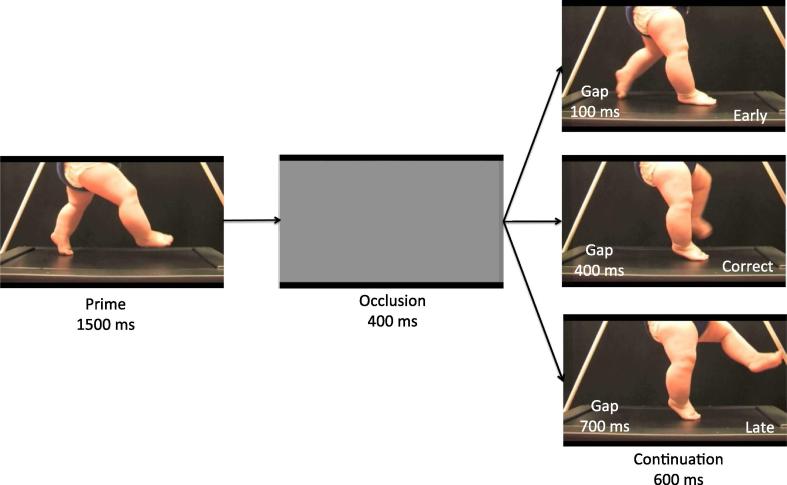
Example still frames of a video from the ‘upright’ condition in Experiment 1.

The stimulus material for the test trials consisted of video clips of six different infants performing stepping actions on an infant treadmill filmed from a sagittal view (see de Klerk, Johnson, Heyes et al., 2015). All stepping actions were rightward movements but as the infants were on a treadmill, there was little horizontal translation. Familiarisation stimuli consisted of three infants performing bouncing actions on the infant treadmill. The video-clips were edited to show only the legs of the infant against a black background to minimize distraction by the face or moving arms. For the inverted condition the videos were flipped along the vertical axis to invert the stimuli without changing the general direction of the movement. This resulted in a total stimulus set of 12 test trials consisting of 6 upright and 6 inverted stepping actions, and 6 familiarisation trials consisting of 3 upright and 3 inverted bouncing actions (see Fig. 1 for still frames of example stimuli from the upright condition).

Stimuli started with a 1500 ms movement prime during which the start of a stepping or bouncing action was presented. Hereafter the action was occluded from view by a light grey occluder for 400 ms. This occlusion duration was chosen based on previous studies with adult participants that showed that predictive tracking accuracy was highest when actions were occluded for 400 ms (Graf et al., 2007, Springer and Prinz, 2010). Immediately after the occlusion, the action either resumed at a frame that corresponded to a time-coherent continuation of the action (i.e., 400 ms after the last frame before occlusion) or at a frame too early or too late in the action sequence (−100 or +700 ms after the last frame before occlusion). Similar time-shifts were used in previous studies with adult participants (Graf et al., 2007, Springer and Prinz, 2010). This resulted in four different trial types: upright correct, upright incorrect, inverted correct, inverted incorrect. In the familiarisation trials, the continuation of the action was always time-coherent. In both the familiarisation and test trials, the action continued for 600 ms after the occlusion before the image froze (see Fig. 1 for the stimuli sequence).

#### 2.1.3 Design and procedure

Infants were seated on their caregiver’s lap in a darkened room at a distance of approximately 60 cm from a 19-inch (4:3 aspect ratio) CRT monitor on which the visual stimuli were presented. Every trial started with a central attention-getter consisting of a colourful, screensaver-like video that was accompanied by a sound. Once the infant oriented towards the screen the experimenter triggered the presentation of a trial. Infants were first presented with 3 upright and 3 inverted familiarisation trials to get accustomed to the trial sequence. Hereafter a maximum of 16 test trials was presented; 4 upright correct, 4 upright incorrect, 4 inverted correct, and 4 inverted incorrect trials. In half of the incorrect continuations the continuation was too early, and in the other half the continuation was too late. Preliminary analyses during pilot testing (N = 6) revealed no looking time differences between the ‘too early’ and ‘too late’ continuations (main effect *F*(1, 4) = 0.965, *p* = 0.382; interaction with trial type (upright or inverted), *F*(1, 4) = 1.033, *p* = 0.367), therefore these two types of ‘incorrect’ continuation were collapsed.

The experimenter, who was hidden behind a curtain, assessed infants’ looking times to the still frame after the correctly or incorrectly continued actions online. Trials ended when the infant looked away from the screen for more than 2 consecutive seconds or when 10 s (in the familiarisation trials) or 20 s (in the test trials) had elapsed. Trials in which infants did not look at the entire sequence of movement prime, occlusion, and continuation were excluded from the analyses. Infants had to have at least 2 valid trials in each of the four test trial types to be included in the analyses (5 infants were excluded for not meeting this criterion). The order of the four trial types (upright correct, upright incorrect, inverted correct, and inverted incorrect) was counterbalanced between infants, and this order was repeated up to four times or until the infant’s attention could no longer be attracted towards the screen.

#### 2.1.4 Data analysis

Looking times to the still frame after the continuation were coded offline by two coders: the first author and a research assistant who was blind to the design and hypothesis of the study. The looking times of half of the infants were coded by both coders and were highly correlated, *r* = 0.967, *p* < 0.001, with a mean absolute difference of 316 ms. Analyses were carried out on the coding of the first author. Only 11 out of the 24 infants had 3 or more valid test trials in all four trial types. Therefore we decided to use the first 2 valid test trials for each trial type for the analyses.

### 2.2 Results

**Fig. 2:**
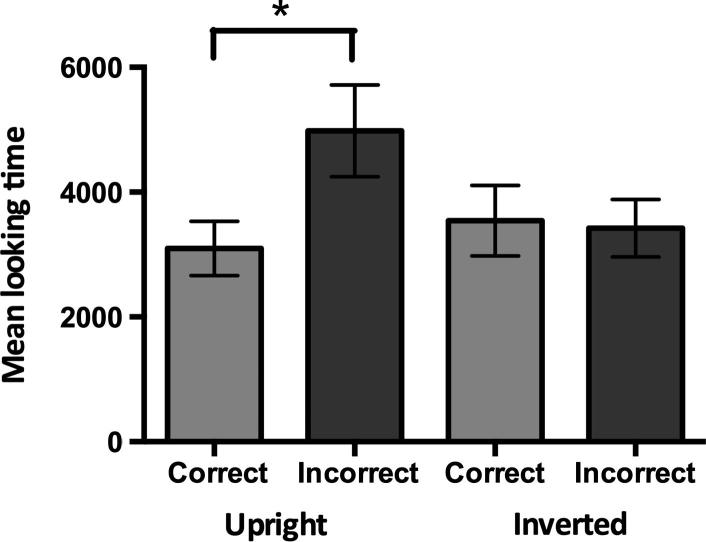
Average looking times to the still frame for the first two trials of each condition. Significant differences between conditions are indicated, ^∗^ *p* < 0.05. Error bars represent SEM.

Analyses were carried out on the mean looking times during the first two valid trials for each of the four trial types (see Fig. 2). Because looking times tend to be distributed log-normally across participants (Csibra, Hernik, Mascaro, Tatone, & Lengyel, 2016), the data were log-transformed prior to parametric analyses. A repeated measures ANOVA with continuation (correct vs. incorrect) and orientation (upright vs. inverted) as within subjects factors demonstrated a main effect of continuation, *F*(1, 23) = 5.702, *p* = 0.026, $\eta_{p}^{2}$ = 0.199. Although the interaction between continuation and orientation was not significant, *F*(1, 23) = 2.283, *p* = 0.144, $\eta_{p}^{2}$ = 0.090, the main effect of continuation, i.e., longer looking times after the incorrect compared to the correct continuations, seemed to be solely driven by the upright condition (see Fig. 2). Planned comparisons indeed demonstrated that infants looked significantly longer after the incorrect compared to the correct continuations of the upright, *t*(23) = 2.461, *p* = 0.022, but not the inverted stepping actions, *t*(23) = 0.210, *p* = 0.836. Non-parametric Wilcoxon tests further supported this finding: infants looked longer after the incorrect compared to the correct continuations of the upright stepping actions, Z = 2.257, *p* = 0.024, but not of the inverted stepping actions, Z = 0.629, *p* = 0.530.

Previous work has shown that infants have a visual preference for upright over inverted biological motion (Fox and McDaniel, 1982, Simion et al., 2008). This raises the concern that infants may have simply attended more to the upright compared to the inverted stimuli, allowing them to distinguish between the incorrect and correct continuations in the former condition only. However, there was no main effect of orientation, *F*(1, 23) = 1.613, *p* = 0.217, demonstrating that infants did not look longer to the upright compared to the inverted stimuli. Additionally, there were no differences in the number of invalid trials (i.e. trials in which infants did not look at the entire sequence of events) between the upright and inverted conditions, *F*(1, 23) = 0.611, *p* = 0.443. These findings suggest that our results were not driven by differences in overt attention to the upright and inverted stimuli.

### 2.3 Discussion

Consistent with our prediction, the looking time results demonstrated that infants were only able to distinguish between the correct and incorrect continuations of the stepping actions that were presented from a visually familiar perspective. The significantly longer looking times after the incorrect continuations of the upright stepping actions demonstrate that infants’ expectations about what the continued action should look like were violated. This suggests that they were able to maintain a representation of the stepping action during the occlusion, which allowed them to predict how the action should continue once the occluder disappeared. As our participants were not able to walk yet, this finding demonstrates that motor experience is not necessary for accurate predictive action tracking. Based on the findings of a previous study, in which we found greater sensorimotor cortex activation after repeated exposure to these infant stepping stimuli (de Klerk, Johnson, Heyes et al., 2015), we were initially expecting that infants might need visual training with the stimuli before they would be able to predictively track the actions. However, instead we found that infants were able to distinguish between the correctly and incorrectly continued actions without receiving any visual training with the stimuli. These findings suggest that infants in our previous study (de Klerk, Johnson, Heyes et al., 2015) were also able to predict the kinematics of the stepping actions even before being familiarised with the stimuli. However, possibly the visual experience that these infants received over the course of that study may have allowed them to make more exact predictions about the stepping actions at post-test, such that even small deviations from these predictions increased the prediction error and hence the amount of sensorimotor alpha suppression (Cross et al., 2012). These findings also suggest that previous visual experience with similar actions might be able to support real-time action prediction. Although the treadmill-elicited infant stepping actions that infants observed in these studies may seem quite different from the walking actions they typically observe in their environment, it has been demonstrated that the EMG pattern and rhythmicity of treadmill-elicited stepping actions in young pre-walking infants (10 weeks to 10 months) show many of the characteristics of adult walking (Yang, Stephens, & Vishram, 1998). Furthermore, research with adult participants has shown that a relatively brief observational training (four times 45 min) can generalise to a large extent to untrained stimuli (Cross et al., 2013). Our results suggest that infants’ extensive observational experience with walking actions may have allowed them to generate expectations about the temporal dynamics of the infant stepping stimuli in the present study. Possibly the activation of motor programs for actions with similar temporal dynamics supported these fine-grained temporal predictions. Experiment 2 investigates this idea.

Our findings seem to be inconsistent with previous eye-tracking studies that found that infants’ ability to predict actions depended on their ability to competently perform the observed actions (Cannon and Woodward, 2012, Cannon et al., 2012, Falck-Ytter et al., 2006, Gredebäck and Kochukhova, 2010, Gredebäck et al., 2009, Kanakogi and Itakura, 2011). The reason for this discrepancy could be that while these previous studies measured infants’ anticipatory saccades, the present study measured looking times. It has been suggested that looking times and anticipatory saccades reflect different types of processing, with the former reflecting post hoc comparisons of expected and observed actions, and the latter reflecting real-time expectations about how the actions will continue (Daum, Attig, Gunawan, Prinz, & Gredebäck, 2012). In most looking time studies, the still image that is presented until the infant looks away indeed contains information that allows the infant to determine whether their expectation was violated or not. In the incorrect trials in the current study, however, the expectation that was violated was a temporal one, and the still image did not provide any clues with regards to whether the continuation of the action had been correct or incorrect. Infants’ expectations could only be violated if they were able to dynamically maintain a representation of the stepping action during the occlusion, allowing them to predict what the action continuation should look like once the occluder disappeared. Therefore, even though we used a ‘post-hoc’ measure, we did measure the result of *real-time* action prediction processes. We propose that the more important difference between the previous anticipatory looking paradigms and the current study is that even though infants needed to have a prediction about how the actions would continue in both cases, the present study did not require infants to generate an immediate behavioural response based on this prediction. While anticipatory looking paradigms require the infants to disengage from the ongoing action and make an anticipatory saccade - which may be more difficult when infants are less familiar with the actions - our looking-time measure only required infants to look away from the static image on the screen.

## 3 Experiment 2: EEG

What neural mechanisms underlie infants’ ability to predictively track actions that are outside their motor repertoire? One possibility is that infants’ previous observational experience with walking actions allowed them to activate motor programs for alternative actions with similar temporal dynamics to support their predictions about how the actions would unfold. This hypothesis is based on the idea that the motor system is thought to be involved in predicting a wide variety of events, ranging from complex human movements that are outside the observers’ motor repertoire (e.g. Cross et al., 2013), to sequences of abstract visual stimuli (e.g. Schubotz & von Cramon, 2003) or the movement of self-propelled objects (e.g. Southgate & Begus, 2013). Thus, contrary to the view that observers need to have access to a *corresponding* functional motor representation for successful action prediction, the motor system can be recruited for any kind of event prediction. We hypothesised that if the sensorimotor cortex is involved in predictive action tracking, independent of motor experience, we should find stronger sensorimotor cortex activation during the occlusion of the visually familiar upright stepping actions that, according to Experiment 1, infants are able to predictively track, than during the occlusion of the visually unfamiliar inverted stepping stimuli that they are unable to predictively track. Based on the idea that perception of dynamic events is always predictive in nature (Wilson & Knoblich, 2005), we expected sensorimotor cortex activation to be present whenever there were events to predictively track. However, our analyses focused on the occlusion period, as we hypothesised that sensorimotor alpha suppression during this period would specifically be related to maintaining a representation of the stepping action while it was no longer visible on the screen, allowing infants to predict how the action should continue once the occluder disappeared.

It has been suggested that when the observer is unable to perform the observed action, he or she may activate the motor program for an action that could bring about similar effects (Csibra, 2007, Schubotz, 2007). In the present experiment kicking might be such a potential alternative action, as seminal work by Esther Thelen and colleagues has demonstrated that the rhythmical kicking actions that pre-walking infants spontaneously perform have a spatial and temporal organisation similar to mature walking (Thelen, Bradshaw, & Ward, 1981). If the infants indeed activated the motor programs for other rhythmical leg actions to facilitate the predictive tracking of the stepping actions, we would expect to see sensorimotor alpha suppression localized to the leg areas (de Klerk, Johnson, & Southgate, 2015).

To investigate these hypotheses, we used electroencephalography (EEG) to measure the attenuation of the sensorimotor alpha rhythm as a measure of sensorimotor cortex activation (Marshall et al., 2011, Southgate et al., 2009, Southgate et al., 2010) while a different group of pre-walking infants observed the upright and inverted stepping stimuli that were employed in Experiment 1.

### 3.1 Method

#### 3.1.1 Participants

The final sample consisted of 16 pre-walking 8-month-old infants (seven females, M = 247 days; range 225–272 days). An additional 23 infants were tested but excluded because they did not provide enough artefact-free trials for analyses due to movement, fussiness, or poor signal quality (N = 22) or technical error (N = 1). The final number of infants included and the percentage of excluded participants are typical of EEG studies with infants (e.g., Marshall et al., 2011, Southgate and Begus, 2013, Southgate et al., 2009, Southgate et al., 2010). All included infants were born full-term, healthy and with normal birth weight.

#### 3.1.2 Stimuli

Infants were presented with the same temporarily occluded upright and inverted stepping actions that were used in Experiment 1 (see Fig. 1). The only difference with Experiment 1 was that the image froze only for 500 ms after the continuation of the action. This allowed us to present more trials in the limited amount of time infants would remain attentive and still during the EEG session.

#### 3.1.3 Design and procedure

The experiment was performed in the same testing environment as Experiment 1. The upright and inverted conditions were presented in a random order. Each trial was preceded by a baseline phase presenting infants with a colourful, screensaver-like video accompanied by sounds. The duration of the baseline phase varied randomly between 1650 and 2000 ms. The recording lasted up to 8 min or until the infant was no longer attending to the videos.

#### 3.1.4 Recording and processing of EEG

EEG was recorded using a 128-channel Geodesic Sensor Net (GSN; EGI Inc, Eugene, Oregon) at 500 Hz sampling rate with respect to the vertex electrode. EEG data were segmented into epochs of 4800 ms length around each trial, consisting of 1000 ms baseline, 1500 ms movement prime, 400 ms occlusion, 600 ms continuation, and 500 ms of still frame, plus a 400 ms buffer on either side of the segment. Trials in which, according to the video recording, the infant did not attend to the screen or made any limb movements were excluded. Furthermore, trials with additional EEG artefacts were rejected based on careful visual inspection. Only infants with at least 8 artefact-free trials per condition were included in the analyses. Infants contributed a mean number of 11.8 artefact-free upright trials (SD = 3.90, range: 8–20 trials) and 11.6 artefact-free inverted trials (SD = 3.91, range: 8–21 trials) to the analyses.

The artefact-free trials were re-referenced to the average EEG activity, and then time-frequency analyses were performed on them by continuous wavelet transform using Morlet wavelets at 1 Hz intervals in the 5–25 Hz range. To eliminate distortion created by the wavelet transform, the first and last 400 ms of each trial were removed after the transformation. Activity in the 6- to 9-Hz-frequency range during 400 ms of the baseline period was subtracted from the 400 ms occlusion period. Average wavelet coefficients were calculated for each infant by taking the mean across the trials. We selected a cluster of electrodes (7, 31, 55, 80, 106 and Cz) over which the sensorimotor alpha rhythm is specifically modulated by leg actions in infants (de Klerk et al., 2015, van Elk et al., 2008). To ensure that any effects were specific to the central leg representation area we also analysed data over a cluster of electrodes over the left sensorimotor area (30, 36, 37, 41, and 42) that was predominantly activated during observation of manual actions in previous infant studies (Southgate and Begus, 2013, Southgate et al., 2009, Southgate et al., 2010). Additionally, to ensure that our data specifically reflected changes in the sensorimotor alpha rhythm and not the more posterior occipital alpha rhythm, we included a cluster of occipital channels as well (electrodes 70, 71, 75, 76, and 83).

### 3.2 Results

A repeated measures ANOVA on alpha suppression during the occlusion with orientation (upright vs. inverted) and channel cluster location (left, central, and occipital) as within subjects factors demonstrated a main effect of orientation, *F*(1, 15) = 5.781, *p* = 0.030, $\eta_{p}^{2}$ = 0.278, a main effect of location, *F*(2, 30) = 9.047, *p* = 0.001, $\eta_{p}^{2}$ = 0.376, and a marginally significant interaction between orientation and location, *F*(2, 30) = 3.273, *p* = 0.052, $\eta_{p}^{2}$ = 0.179. Bonferroni-corrected post hoc *t*-tests demonstrated significantly weaker alpha suppression over the occipital channel cluster compared to the left channel cluster, *p* = 0.001.

**Fig. 3:**
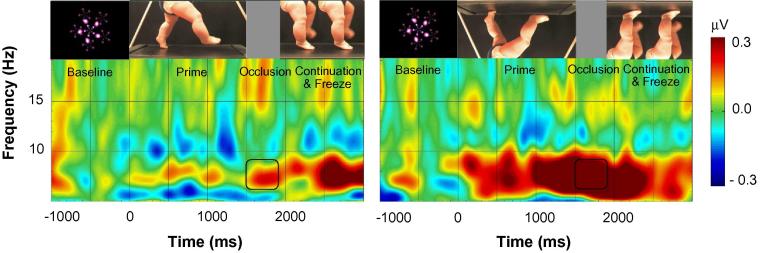
Time-frequency plots demonstrating the changes in sensorimotor alpha amplitude (6–9 Hz) during the observation of the upright and inverted stepping actions over the leg area. More negative amplitudes indicate more sensorimotor alpha suppression. The zero point indicates the start of the video. Black rectangles indicate the time and frequency range over which statistics were computed.

Follow-up repeated measures ANOVAs were conducted separately for each channel cluster. These analyses demonstrated a significant effect of orientation for the central (leg-area) channels, *F*(1, 15) = 9.252, *p* = 0.008, $\eta_{p}^{2}$ = 0.381, which did not result from differences in sensorimotor alpha activation during the baseline period, *F* (1, 15) = 0.129, *p* = 0.725. This finding demonstrates that infants recruited their sensorimotor cortex significantly less (as evidenced by an increase in sensorimotor alpha amplitude; see Fig. 3) during the occlusion of the visually unfamiliar inverted stepping actions than during the occlusion of the familiar upright stepping actions. Non-parametric Wilcoxon tests further supported this finding: infants showed less sensorimotor alpha suppression during the occlusion of the inverted compared to the upright stepping stimuli, Z = 2.689, p = 0.007. There was no effect of orientation on sensorimotor alpha suppression measured over the left and occipital channel clusters, all *p*s > 0.305, demonstrating that these results were specific to the central leg areas and independent of the occipital alpha rhythm.

As can be seen in Fig. 3, the effects of orientation over the leg-area were driven by a significant de-activation in the inverted condition instead of by significant activation in the upright condition. Possibly this absence of activation in the upright condition was driven by sensorimotor alpha suppression being present during the baseline period in both conditions. To determine whether this indeed was the case, we would ideally have used another baseline period during which there was no suppression present (e.g., activation in response to observing a blank screen). Unfortunately, we did not have such a period in our recording. Rather, we decided to use as an alternative baseline the time when the image of the upright or inverted legs was frozen on the screen, because during this time there were no longer any movements to predictively track. Thus, the last 400 ms of the still frame at the end of each trial, during which there was no alpha-suppression in either of the conditions, was adopted as an alternative baseline. Activity in the 6- to 9-Hz frequency range during this 400 ms baseline period was subtracted from the 400 ms occlusion period. Repeated-measures analyses on sensorimotor alpha suppression calculated with this new baseline demonstrated highly similar results. Again, there was a significant main effect of location, *F*(2, 30) = 16.337, *p* < 0.001, $\eta_{p}^{2}$ = 0.521, and a marginally significant interaction between orientation and location, *F*(2, 30) = 2.812, *p* = 0.076, $\eta_{p}^{2}$ = 0.158. Follow-up repeated measures ANOVAs again demonstrated a significant effect of orientation over the central channels, *F*(1, 15) = 4.934, *p* = 0.042, $\eta_{p}^{2}$ = 0.248, which did not result from differences in sensorimotor alpha activation during the baseline period, *F* (1, 15) = 0.863, *p* = 0.368.

**Fig. 4:**
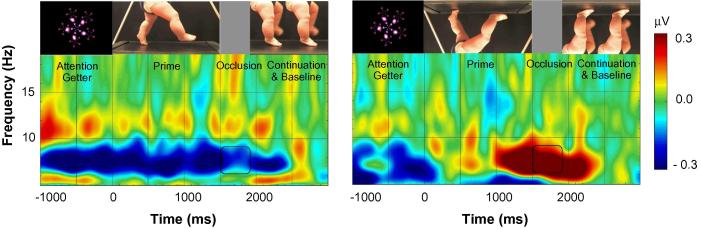
Time-frequency plots demonstrating the changes in sensorimotor alpha amplitude (6–9 Hz) during the observation of the upright and inverted stepping actions over the leg area. The baseline was taken from the last 400 ms of the still frame of the upright or inverted legs. More negative amplitudes indicate more sensorimotor alpha suppression. The zero point indicates the start of the video. Black rectangles indicate the time and frequency range over which statistics were computed.

Thus, using the alternative baseline at the end of the trial, we found that infants recruited their sensorimotor cortex (as evidenced by a decrease in sensorimotor alpha amplitude; see Fig. 4) significantly more during the occlusion of the visually familiar upright stepping actions than during the occlusion of the unfamiliar inverted stepping actions. Non-parametric Wilcoxon two-tailed tests further supported this finding: infants showed more sensorimotor alpha suppression during the occlusion of the upright compared to the inverted stepping stimuli, *Z* = 2.327, *p* = 0.020. Again we found no effect of orientation on sensorimotor alpha suppression measured over the left and occipital channel clusters, all *p*s > 0.360, demonstrating that the results were specific to the central leg areas, and independent of the occipital alpha rhythm. As can be seen in Fig. 4, in the upright condition there was a similar amount of sensorimotor alpha suppression during the screensaver-like baseline and during the movement prime and occlusion. This finding suggests that infants might have been predicting that there would be upright stepping actions before the onset of each trial (cf., Southgate et al., 2009). While in the upright condition this prediction was maintained during the movement prime and occlusion period, in the inverted condition infants’ predictions were violated resulting in de-activation of the sensorimotor cortex. However, despite the significant difference between the two conditions during occlusion, the sensorimotor alpha suppression in the upright condition was not significantly different from baseline.

### 3.3 Discussion

Infants produced significantly greater sensorimotor cortex activation during the occlusion of the visually familiar upright stepping actions, that infants in Experiment 1 *could* predict, compared to during the occlusion of visually unfamiliar inverted stepping actions that infants in Experiment 1 *could not* predict. This finding is consistent with previous studies that demonstrated more motor system involvement during the observation of visually trained versus untrained stimuli (Burke et al., 2010, Cross et al., 2009, de Klerk et al., 2015, Frey and Gerry, 2006) and supports the idea that observational experience might shape the sensorimotor regions of the brain in a similar manner as physical experience does (Burke et al., 2010, Cross et al., 2009). Additionally, these findings provide further support for the idea that motor expertise is not a prerequisite for motor system activation during action prediction (Abreu et al., 2012, Schubotz, 2007, Southgate and Begus, 2013). The finding that the effects were specific to the central leg areas is consistent with the idea that infants may have activated motor programs for other leg actions, such as kicking, that have similar temporal dynamics to walking (Thelen et al., 1981). However, as the sensorimotor alpha suppression in the upright condition was not significantly different from baseline, these results preclude us from drawing firm conclusions about the role of the motor system in predicting human actions outside the infant’s motor repertoire. A possible explanation for the absence of significant suppression from baseline is that fact that the stepping actions were presented as isolated limbs on a video display, as previous research has shown that actions observed in live settings elicit greater sensorimotor cortex activation (Ruysschaert et al., 2013, Shimada and Hiraki, 2006). Although previous sensorimotor alpha studies have successfully used video stimuli in which only a part of the actor’s body was visible (de Klerk et al., 2015, de Klerk et al., 2015, Southgate et al., 2009, Southgate et al., 2010, Southgate and Begus, 2013), it is possible that seeing the actor’s whole body is particularly important to obtain activation that is significantly different from baseline, and future studies are needed to investigate this possibility.

The de-activation of the sensorimotor cortex during the observation of the highly unfamiliar inverted stepping actions in Experiment 2 does not fit with the results of previous studies that demonstrated *greater* activation of the motor system in infants and adults during the observation of unfamiliar actions (Cross et al., 2013, Grossmann et al., 2013, Stapel et al., 2010). Possibly, some minimal level of visual familiarity with the observed actions is required before infants attempt to generate action predictions. Alternatively, after infants failed to predictively track the inverted actions during the first few trials they may have given up trying to generate predictions for this type of stimulus, resulting in de-activation of the sensorimotor cortex. However, these interpretations remain speculative and future research is needed to systematically investigate the relationship between motor system activation during action prediction and visual and motor familiarity with the observed actions in infancy (Grossmann et al., 2013).

## 4 General discussion

In recent years, much attention has been focused on the notion that having access to a corresponding functional motor representation is a crucial prerequisite for the ability to predict observed actions (e.g. Cannon and Woodward, 2012, Cannon et al., 2012, Falck-Ytter et al., 2006, Gredebäck and Kochukhova, 2010, Gredebäck et al., 2009, Kanakogi and Itakura, 2011, Stapel et al., 2016). As a result of this focus on the role of motor experience in action prediction, very little research has been conducted to investigate alternative mechanisms through which infants can understand and predict others’ actions while their motor repertoire is still developing. The results of the present study show that infants are able to make surprisingly fine-grained predictions about the kinematics of human actions that are outside their motor repertoire, and that the sensorimotor cortex may play a role in this predictive tracking process. Pre-walking infants distinguished between correctly and incorrectly continued stepping actions that were presented from a visually familiar perspective only, suggesting that visual experience alone may be sufficient to support successful predictive action tracking. These findings are consistent with previous studies that have demonstrated that infants can predict events for which they do not have access to a corresponding functional motor representation (i.e., non-human actions such as self-propelled objects and claws) (Biro, 2013, Southgate and Begus, 2013) and that suggest that observational experience can facilitate the activation of motor representations for unfamiliar actions (Boyer & Bertenthal, 2016). The present study extends these results by demonstrating that infants can generate real-time action predictions for *human* actions that are not yet part of their motor repertoire. Taken together, these findings provide evidence against the idea that predictive tracking of human actions requires motor experience with the observed actions.

Motor activation during action prediction is thought to be the result of an action reconstruction process during which observed actions are first interpreted at some level of visual or conceptual analysis, after which they are transformed into motor code allowing the observer to predictively track the visual consequences of the action (Wolpert and Flanagan, 2001, Wolpert and Ghahramani, 2000). When the observer is unable to perform the observed action, he or she may activate the motor program for an action that could bring about similar effects (Csibra, 2007, Schubotz, 2007). This model suggests that infants in the current experiment may have employed a mid-level visual interpretation of the stepping actions (e.g., the left leg is moving upwards and forwards with a certain pace), which the motor system attempted to approximate using the motor programs available to the infant. This internal motor representation in turn would have allowed infants to maintain a representation of the stepping action during the occlusion, enabling them to predict what the action should look like once the occluder disappeared.

The model by Schubotz (2007) proposes that observers activate the motor program for an action that most closely matches the relevant properties of the observed action. The finding that the sensorimotor alpha effects were localised to the central leg areas suggests that infants may have activated the motor program for other rhythmical leg movements, such as kicking, to predict the observed stepping actions. However, as the sensorimotor alpha suppression during the observation of the upright stepping actions was not significantly different from baseline, the results of Experiment 2 need to be interpreted with caution. A possible explanation for the absence of significant suppression is that the actions were presented on video rather than live, as previous studies have found stronger sensorimotor alpha suppression for live presentations (Ruysschaert et al., 2013, Shimada and Hiraki, 2006). Nevertheless, the current findings are inconclusive, and do not allow us to draw firm conclusions about whether or not motor mechanisms play a functional role in the predictive tracking of human actions that are outside the infant’s motor repertoire. We also cannot exclude the possibility that infants relied on perceptual expectations without involvement of the motor system to differentiate between the correctly and incorrectly continued stepping actions. Future studies will need to address this question more directly, preferably by simultaneously obtaining behavioural measures of predictive action tracking and neurophysiological measures of motor system activation to investigate whether there is a functional link between the two in infancy.

Based on the action reconstruction account discussed above, one would expect that prediction accuracy will be modulated by the similarity between the motor commands activated in the observer and those needed to perform the observed action, and studies with adult participants have indeed confirmed this is the case (e.g. Aglioti et al., 2008, Diersch et al., 2012, Knoblich and Flach, 2001, Knoblich et al., 2002). Therefore with increasing competency in performing actions, the accuracy of infants’ action predictions is also expected to increase (e.g. Kanakogi and Itakura, 2011, Stapel et al., 2016). Although this relationship between action expertise and prediction is well-documented, it is important to note that experience only modulates, but does not determine, the ability to predict actions (Schubotz, 2007).

We found that pre-walking infants were able to distinguish between the correct and incorrect continuations of stepping actions that were presented in a visually familiar orientation. Although infants did not receive any visual training with the stimuli, it is possible that they were able to learn about the temporal dynamics of the stepping actions over the course of the experiment. However, the difference in looking times between the correctly and incorrectly continued upright stepping actions was already marginally significant when only the first valid trial of each condition was included in the analysis (*t*(23) = 1.855, *p* = 0.076), and it seems unlikely that infants received sufficient experience with the stepping stimuli during those first few trials for visual learning to take place. A more plausible possibility is that even though the infants did not have previous experience with observing infant stepping actions, they were able to generalise from their extensive experience with observing others’ walking actions to predict the kinematics of the infant stepping actions. Evidence from a study on the effects of visual training on predictive action tracking in adult participants supports the idea of such generalisation from ‘trained’ to ‘untrained’ stimuli (Cross et al., 2013).

However, this raises the question of why infants were unable to generalise from their observational experience with upright walking to predictively track the inverted stepping actions. Even though the low-level visual and motion features are highly similar for upright and inverted actions, infants do not have any experience with observing upside down actions. Because of this lack of experience, and/or because of the fact that inverted actions violate gravitational constraints, infants may have been unable to recognise the inverted stimuli as a familiar human shape (Bertenthal et al., 1987, Troje and Westhoff, 2006). The finding that infants were unable to predictively track the inverted actions is consistent with previous studies with adult participants in which real-time action prediction also broke down when actions were inverted (Graf et al., 2007, Sparenberg et al., 2012). One final possibility that would explain both the adult work and the present findings is that humans may possess an inborn ability to represent the temporal dynamics of upright biological motion, and future research with newborn infants could shed light on this question.

### 4.1 Conclusions

Although active action experience undeniably provides infants with a rich source of information about human actions, the results of the current study demonstrate that it is not necessary for successful predictive action tracking. Instead, our findings suggest that infants can generate fine-grained temporal predictions about the kinematics of others’ actions, possibly based on previous visual experience, without being able to map the observed actions onto a motor representation for the same action. The focus on the relationship between infants’ ability to perform and predict actions may have led researchers to overlook other ways in which infants can learn about the actions of the people around them. Considering the important role real-time action prediction plays in joint action, cooperation, and collaboration (Sebanz & Knoblich, 2009), future studies should further investigate, using measures that do not require an immediate behavioural response, the mechanisms by which infants can understand and predict others’ actions while their motor repertoire is still developing.

## Footnotes

1 In this paper, the terms ‘predictive action tracking’ and ‘real-time action prediction’ refer to mechanisms by which the kinematics or movement paths, rather than the goal or the outcome, of observed actions are tracked and predicted in real time.
